# Ten-year audit of Lichtenstein hernioplasty under local anaesthesia performed by surgical residents

**DOI:** 10.1186/1471-2482-10-24

**Published:** 2010-08-04

**Authors:** Hannu Paajanen, Riitta Varjo

**Affiliations:** 1 Department of Surgery, University Hospital of Kuopio, Puijonlaaksontie 2, 70210 Kuopio, Finland; 2 Department of Surgery, Central Hospital of Mikkeli, Porrassalmenkatu 35-37, 50100 Mikkeli, Finland

## Abstract

**Background:**

To analyse in a prospective trial the long-term results of Lichtenstein hernioplasty performed by surgical trainees.

**Methods:**

Training of tension-free Lichtenstein hernia operation was started in our ambulatory unit as an outpatient procedure under local anaesthesia in 1996. After performing 36 teaching operations together with residents and their supervising specialist, 281 patients were operated during 1996-2000 either by one senior consultant (n = 141) or by 12 surgical trainees (n = 140). After 10 years, 247 (88%) patients were available for the long-term assessment.

**Results:**

After one month postoperatively, the rate of wound infections (consultant 1.1%, residents 0.7%) and hematomas (consultant 1.1%, residents 3.0%) were low and not related to surgeon's training level (ns). Only 6 (2.1%) clinically evident recurrences were found after 10 years: two after specialist repair and four after trainee repair (ns). Although one third of the patients reported some discomfort after 3 and 10 years, 93-95% of the patients were very satisfied with the operation, with no statistical difference between the surgeons.

**Conclusion:**

Ambulatory open mesh repair under local anaesthesia was a safe operation and the long-term results were acceptable among the patients operated by surgical trainees.

## Background

Inguinal hernias occur in about 15% of adult men and hernioplasty is the most common surgical procedure performed by general surgeons [1]. Approximately 11 000 inguinal hernioplasties are performed each year in Finland, over 80 000 operations in England and over 800 000 in the United States [1-3]. In Scandinavian countries, the majority of groin hernias are currently operated in ambulatory surgical units. About 20% of groin hernia repairs are done due to recurrences and only 4% as emergency [1-3]. The economical impact of groin hernia surgery is high on the health care system.

There is strong evidence that surgeon's case volume, hospital volume and specialisation improve the outcome of many major surgical procedures, such as coronary artery bypass, gastrectomy, esophagectomy, pancreaticoduodenectomy and rectal cancer surgery [4,5]. The role of specialist centers in more common surgical operations, such as colon resections or inguinal hernioplasties, is not so clear [3,6]. Although inguinal hernioplasty is one of the first operations performed by surgical residents, only few studies have compared the immediate results between residents and their consultant [7-11]. The reliable recurrence rate of inguinal hernioplasty needs over 5 years of follow-up, and there are not available such long-term studies between residents and attending surgeons so far.

Lichtenstein hernioplasty is a tension-free technique, which uses polypropylene mesh to support the inguinal muscular layers [12]. Its learning curve may be shorter than traditional groin hernioplasties, and therefore Lichtenstein procedure has rapidly increased as a primary operation in inguinal hernia in many countries. Under local anaesthesia, it can be performed as a rapid outpatient procedure with cost savings [13]. The present study was designed as a quality control audit in the surgical training program for this common surgical procedure. The main interest was whether well-trained surgical residents are able to perform Lichtenstein operation with an acceptable immediate and long-term outcome compared to the experienced specialist in hernia surgery.

## Methods

**Figure 1:**
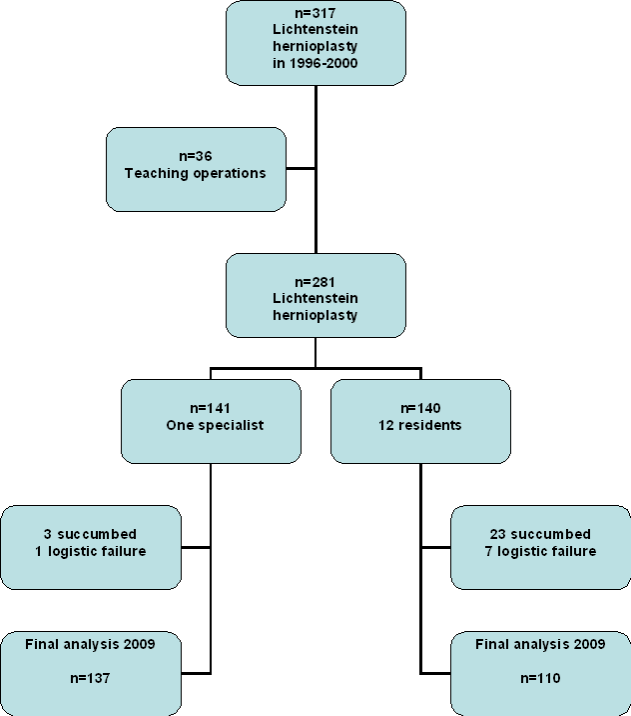
**Flow chart of the study**.

This was a comparative prospective trial of 317 adult patients with inguinal hernia. The patient characteristics and types of hernia are presented in Table 1. Fourteen patients (4.4%) had recurrent hernia. No mesh implantation had been used earlier to any patients. The exclusion criteria were femoral hernia, emergency operation, allergy to polypropylene or patient's refusal to participate in the study. One consultant surgeon or twelve residents of general surgery (3-4 years of residency) performed all operations. The consultant operated annually over 200 inguinal hernias using both open mesh and laparoscopic techniques. The design and conduct of the study is presented in Figure 1. After 36 teaching operations together with residents and their consultant, 281 consecutive inguinal hernioplasties were enrolled of the study: 141 patients were operated in local anaesthesia by one consultant and 140 operated by 12 residents (Figure 1). The first three operations of the residents were supervised by the scrubbed consultant surgeon, and thereafter the consultant was on call and advised if necessary. The trainees operated about 10 patients during their 3-month rotation in the ambulatory unit. A secretary of the ambulatory unit scheduled equally the operations for surgeons, and they performed the procedures during their daily rotations in the unit. For ethical reasons, no sealed envelopes or computer programs were used in the patient' selection between trainees and surgeon. Patients fulfilling the day-case surgery criteria received written and oral information about the aims and content of the study in accordance with the Helsinki Declaration. The staff of our day-case surgery told to the patient that the operation is performed by the attending surgeon of the day (either resident or specialist). The patients knew that they were part of the trial, and an informed consent was signed. The ethics committee in our hospital approved the study protocol.

**Table 1:** Initial operative data of 281 patients undergoing Lichtenstein hernioplasty in local anesthesia during 1996-2000.

	Specialist (%)	Residents (%)	p
	n = 141	n = 140	
Male/female	137/4 (97/3)	136/4 (97/3)	ns
Mean age ± SD (range)	54 ± 15 (17 - 83)	53 ± 12 (19 - 80)	ns
Body mass index ± SD	24 ± 3.2	25 ± 3.0	Ns
Lateral/medial hernia	90/51 (64/36)	73/67 (52/48)	ns
Size of the defect (cm)			
< 1.5	30 (21)	28 (20)	ns
1.5-3	70 (50)	66 (47)	ns
> 3.0	41 (29)	46 (33)	ns
Right/left sided	62/79 (44/56)	63/77 (45/55)	ns
Mean operative time ± SD	39 ± 13 min	62 ± 18 min ***	p < 0.0001
Wound infections	2 (1.1)	1 (0.7)	ns
Wound hematoma	2 (1.1)	4 (3.0)	ns

* includes 36 teaching operations

Our hospital is a non-university teaching hospital with 6-8 surgical residents working at the same time. The annual number of inguinal hernioplasties has varied from 180 to 200 per 100 000 inhabitants. The tension-free Lichtenstein technique was started in January 1996 in our new ambulatory unit. The procedure was always performed under local infiltration anaesthesia as a rapid outpatient surgery using 9 × 13 cm polypropylene mesh (Premilene, B. Braun AG, Germany). The sac of the indirect hernia was either resected or just inverted into the abdomen [12,14]. If the hernia sac was large and direct, it was inverted with absorbable 2-0 absorbable sutures. The inguinal nerves were tried to identify and save if possible. We did not try to identify the three inguinal nerves systematically at operation nor record the nerve identification. The mesh was trimmed and placed between the conjoint tendon, inguinal ligament, pubic bone and external oblique aponeurosis [12,15]. Mesh was always fixed with 3-0 absorbable Dexon^® ^(United States Surgical, Norwalk, CT) sutures. Local infiltration anaesthesia was a 1:1 mixture of bubivacaine (Marcain 5 mg/ml, AstraZeneca, UK) and Citanest-adrenalin (10 mg/ml + 5 μg/ml, AstraZeneca, UK) with an average total volume of 40-50 ml. After surgery, the patient was followed up for 60 - 120 minutes to observe possible wound hemorrhage and then discharged. No prophylactic antibiotics were used. A 0.5-1.0 mg bolus of intravenous alfentanil was given (Rapifen, AstraZeneca, UK), if the patient felt pain during the operation. The same standardized postoperative instructions of our ambulatory unit were given to all patients allowing normal daily activities after operation.

The patient characteristics, type of hernia, operation time and wound complications were recorded by an independent research nurse. Operative time was recorded from infiltration of local anaesthetic to skin closure. The short-term outcome was evaluated 1 month post-operation. The long-term results (3 and 10 years) were asked by using the questionnaire and clinical examination. The questions were based on the study of the Danish Hernia Database [16]. The questionnaire included data of recurrence, pain in the last month at rest and during physical exercise, pain scores (VAS 0-10), testicular pain, need of pain-relieving medications, limitations in work or leisure-time activities, feeling of foreign body in the groin and overall satisfaction with the operation. The questionnaire and clinical examination was performed in 2002 and 2009. If the patient told that the hernia had recurred or that there were problems with the operated area, a physical and ultrasound examination was performed to rule out a recurrent hernia or the etiology of chronic pain. Both ultrasound examination and operative findings during re-surgery were used to confirm recurrences of hernia. Out of 281 patients, only 247 were available for the final analysis in 2009. The patients of the original groups were dropped because they could not be contacted or they were deceased (Figure 1).

The data analysis was carried out using Statistical Package for the Social Sciences (SPPS) for Windows, version 14.0 (SPSS, Chicago, Illinois, USA). The statistical evaluation was performed with a Student's t test for paired values and χ-2 test with Yates correction between the groups. P < 0.05 was regarded as significant for both tests.

## Results

The patient characteristics were similar in both groups (Table 1). Mean operative time was shorter with a consultant than with a resident (p < 0.0001). There were no differences in the number of wound complications between the consultant and the trainees after 1 month post-operation (Table 1).

Chronic pain sensations and patient's compliance to the surgery was asked for the first time after the mean follow-up of 3 years (Table 2). One fourth of the patients announced some degree of pain in the operated area with no difference in the training level of surgeon. Only 3-4% of the patients needed occasionally pain-relieving drugs. Chronic pain was so severe in 6 patients (2.1%), that local corticosteroid injections had been used to reveal discomfort. We did not find any relation of chronic pain to nerve status at operation. Over 90% of patients felt that the operating field had healed well. The same percentage of patients was very satisfied with the day-case surgery and they would come again if necessary (Table 2). Three of the recurrences appeared in the medial border of the mesh near the pubic bone, one through a too wide external ring and one through a femoral canal. All recurrences were confirmed by re-operation. Every tenth patient felt the sensation of a foreign body in the groin area.

**Table 2:** Incidence of chronic pain and recurrences 3 years after Lichtenstein operation.

	Specialist (%)	Residents (%)
	n = 141	n = 140
Chronic groin pain	34 (24)	33 (24)
Testicular pain	19 (13)	11 (7.8)
Need pain-relieving medications?	5 (3.5)	6 (4.2)
Are you satisfied with the operation?	134 (95)	128 (91)
Number of recurrences	2 (1.4)	3 (2.1)
Feeling of foreign body	14 (9.9)	18 (12.8)

The long-term outcome after 10 years did not differ much from the 3 years results (Table 3). About 10% of the patients felt still the sensation of a foreign body in the groin area, and 25-30% felt some discomfort or pain at rest or during daily activities, but usually this was not disturbing. Again, there were no marked differences between the surgeon's groups (Table 3). The number of recurrence was 6/281 (2.1%) during the 10 years follow-up with no statistical difference between the surgeon groups. Chronic pain in the long-term follow-up was also measured by using a visual analogue scale. Usually the pain response was between 0-6 (mean 0.31 ± 1.0) at rest and slightly higher (mean 1.0 ± 1.8) during physical exercise with only minor non-statistical differences between the surgeons (Table 4). After 10 years of Lichtenstein hernioplasty, 3 patients with VAS scale over 5 were also treated by local infiltrations of corticosteroids. Usually corticostreoids caused some relief, but did not abolish chronic pain. During 10 years of follow-up, no patients were re-operated due to chronic pain.

**Table 3:** Outcome of patients after 10 years post-operation.

		Specialist (%)	Residents (%)
		n = 137	n = 110
Feeling of foreign body		12 (8.8)	16 (15)
Has anything been harmed from the mesh?		2 (1.5)	3 (2.7)
Are your testicles normal?		126 (92)	100 (91)
Scar discomfort:	No	99 (72)	70 (64)
	At rest	2 (1.5)	5 (4.5)
	During movement	26 (19)	26 (26)
	Both	10 (7.3)	6 (5.5)
Need pain-relieving medications?		3 (2.2)	4 (3.6)
Are you satisfied with the operation?		130 (95)	102 (93)
Number of recurrences		2 (1.5)	4 (3.6)

**Table 4:** Pain after 10 years measured by visual analoque scale (vas 0 -vas 10) at rest and during physical exercise.

	Number of patients	
	
	Specialist (%)	Resident (%)
	n = 137	n = 110
At rest:		
Vas 0	124 (91)	99 (90)
Vas 1	3 (1.5)	1 (0.9)
Vas 2	3 (2.2)	1 (0.9)
Vas 3	2 (1.5)	4 (3.6)
Vas 4	3 (2.2)	4 (3.6)
Vas 5	0	1 (0.9)
Vas 6	2 (1.5)	0
During physical exercise:		
Vas 0	100 (73)	75 (68)
Vas 1	6 (4.3)	5 (4.5)
Vas 2	8 (5.8)	11 (10)
Vas 3	5 (3.6)	9 (8.1)
Vas 4	8 (5.8)	3 (2.7)
Vas 5	5 (3.6)	4 (3.6)
Vas 6	3 (2.2)	1 (0.9)
Vas 7	0	1 (0.9)
Vas 8	1 (0.7)	1 (0.9)
Vas 10	1 (0.7)	0

## Discussion

Our results indicated that properly trained surgical residents are able to perform Lichtenstein hernioplasty without compromising patient's care and long-term outcome. This is an important finding considering quality control and economical views because the surgeon is the most important variable that influences surgical outcome [4]. The influence of training and experience on the outcome was reflected only by the shortening of operating time, but not the other outcome parameters. Our results are in line with Cueto Rozon and co-workers from France, who concluded that Lichtenstein hernia repair may be performed alone by residents if a precise teaching organization by experimented surgeon is available [8]. This conclusion is not in accordance with Wilkiemeyer and co-workers, who reported that open hernia repairs performed by junior residents were associated with higher recurrence rates than those repaired by more trained surgeons [11]. The recurrence rate in their study varied from 1.1 to 7% in 2 years of follow-up. As overall, complication rates for the open procedures were also much higher than in our study [11]. Our residents were already well-experienced to perform independently soft-tissue surgery, which may explain the different results between the present study and that of Wilkiemeyer and co-workers [11]. A properly allocated and powered randomized study between residents and specialists would be presently difficult to run, because nowadays patients demand always the best possible surgeon to operate their hernias.

Inguinal hernias are so common in population that centralization into the specific hernia centres in Europe has not been carried out. In the United States, the results of such specialist clinics have been encouraging. For example, recurrences between 0 and 1% and infections between 0 and 5% have been reported [12,17,18]. The results of non-specialist hospitals have not been as good reporting the recurrence rates between 4-8% [3,10,19,20]. Our results indicate that open tension-free technique is well suited for smaller community-based and regional hospitals yielding good immediate and long-term results. Inguinal hernioplasty is an ideal operation to teach inguinal anatomy and soft-tissue surgery because the regional anatomy has been well described and the repair techniques are well outlined and reproducible. Our results encourage to perform Lichtenstein hernioplasty safely in general hospitals by well-supervised trainees. This may indicate that the learning curve of Lichtenstein hernioplasty is relatively short and the procedure is simple enough to be part of the surgical training programs [8,9,21].

Chronic pain after inguinal hernia repair was also noticed in the present study. Pain has been reported to occur in between 10-30% of the patients after a groin hernia repair [16,22]. The present study indicated that although 25-30% of the patients reported some pain sensations afterwards in the groin, this was mild in nature since over 90% were very satisfied with the operation. Furthermore, only 7/281 patients used occasionally pain-relieving drugs. We used local corticosteroid injections in 6 patients after 3 years and 3 patients even after 10 years to temporarily reveal pain. No patients were re-operated due to chronic pain, and no meshes were removed, although this may help in some cases [23]. It is now evident, that nerve identification shows a negative correlation with chronic postoperative pain [24]. Ten years ago we tried to save inguinal nerves if possible, but we did not systematically identify or record the nerves. Our operative policy has always been to save the nerves if possible and not to cut them routinely. Neuropathic pain may be disabling and an unfortunate complication, which should be avoided by using a careful operative technique. Our recent register-based study indicated that also in Finland chronic pain is the most frequently observed long-term complication of hernia surgery [2]. In this register-based study, the reported severe complications related to inguinal hernia surgery were not increased in the operations performed by residents [2].

The patient selection is of utmost importance to get favourable results in the inguinal hernia surgery performed by residents. At the time of present study, two thirds of the inguinal hernia surgery was not performed in the ambulatory unit. Therefore some selection bias was happened to explain the good results. Our current policy is to operate all patients suitable for day-case surgery using Lichtenstein technique under local anaesthesia. The per cent of Lichtenstein operations under local anaesthesia is currently over 80% of all groin hernia surgery. Local anaesthesia in primary inguinal hernia repairs should be the method of choice [25]. Indications for laparoscopic hernioplasty are occasionally bilateral hernias, complicated recurrences and a suspicion of incipient hernia.

## Conclusion

Our experience is that open tension-free mesh technique in local anaesthesia is simple enough to be learned in general surgical training.

## Competing interests

The authors declare that they have no competing interests.

## Authors' contributions

HP carried out the design of the study, the data analysis and drafted the manuscript. He was also the senior surgeon operating the patients. RV collected the operative data and postoperative follow-up.

Both authors have read and approved the final manuscript.

## Pre-publication history

The pre-publication history for this paper can be accessed here:

http://www.biomedcentral.com/1471-2482/10/24/prepub
